# Magnesium-Doped Sr_2_(Fe,Mo)O_6−δ_ Double Perovskites with Excellent Redox Stability as Stable Electrode Materials for Symmetrical Solid Oxide Fuel Cells

**DOI:** 10.3390/membranes12101006

**Published:** 2022-10-18

**Authors:** Kun Zheng, Jakub Lach, Hailei Zhao, Xiubing Huang, Kezhen Qi

**Affiliations:** 1 Department of Hydrogen Energy, Faculty of Energy and Fuels, AGH University of Science and Technology, al. A. Mickiewicza 30, 30-059 Krakow, Poland; 2 AGH Centre of Energy, AGH University of Science and Technology, ul. Czarnowiejska 36, 30-054 Krakow, Poland; 3 School of Materials Science and Engineering, University of Science and Technology Beijing, Beijing 100083, China; 4 Beijing Key Lab of New Energy Materials and Technology, Beijing 100083, China; 5 College of Pharmacy, Dali University, Dali 671000, China

**Keywords:** Mg-doped Sr_2_Fe_1.2_Mg_0.2_Mo_0.6_O_6−δ_ and Sr_2_Fe_0.9_Mg_0.4_Mo_0.7_O_6−δ_ double perovskites, excellent redox-stable electrode materials, thermal expansion, oxygen content as a function of temperature, transport properties, symmetrical solid oxide fuel cells

## Abstract

In this work, magnesium-doped Sr_2_Fe_1.2_Mg_0.2_Mo_0.6_O_6−δ_ and Sr_2_Fe_0.9_Mg_0.4_Mo_0.7_O_6−δ_ double perovskites with excellent redox stability have been successfully obtained. The physicochemical properties including: crystal structure properties, redox stability, thermal expansion properties in oxidizing and reducing conditions, oxygen content as a function of temperature and transport properties, as well as the chemical compatibility with typical electrolytes have been systematically investigated. The in situ oxidation of reduced samples using high-temperature XRD studies shows the crystal structure of materials stable at up to a high-temperature range. The in situ reduction and oxidation of sinters with dilatometer measurements prove the excellent redox stability of materials, with the thermal expansion coefficients measured comparable with electrolytes. The oxygen nonstoichiometry δ of compounds was determined and recorded in air and argon up to 900 °C. Sr_2_Fe_1.2_Mg_0.2_Mo_0.6_O_6−δ_ oxide presents satisfactory values of electrical conductivity in air (56.2 S·cm^−1^ at 600 °C) and reducing conditions (10.3 S·cm^−1^ at 800 °C), relatively high coefficients *D* and *k*, and good ionic conductivity (cal. 0.005 S·cm^−1^ at 800 °C). The stability studies show that both compounds are compatible with Ce_0.8_Gd_0.2_O_1.9_ but react with the La_0.8_Sr_0.2_Ga_0.8_Mg_0.2_O_3−d_ electrolyte. Therefore, the magnesium-doped double perovskites with excellent redox stability can be potentially qualified as electrode materials for symmetrical SOFCs and are of great interest for further investigations.

## 1. Introduction

Energy generation by the combustion of fossil fuels (coal, oil, and their derivatives) brings various serious environmental issues, and the depletion of these fuels urgently requires the development of new alternative clean and green energy sources. Solid Oxide Fuel Cells (SOFCs) are among the most promising technologies for the production of electricity and heat from traditional and renewable energy sources [1,2,3,4,5,6,7,8]. The main advantage of SOFCs is their high efficiency and fuel flexibility. They can also significantly reduce CO_2_ emissions and other harmful gases (NO_x_, SO_x_, CO) when compared to traditional combustion technology. However, when the mostly available carbon-containing cheap fuels are applied in SOFCs, carbon deposition over the anode occurs and, in consequence, significantly decreases the power output. Those fuels usually contain a small amount of sulfur, which causes the sulfur poisoning problem (even at a level of several ppm of H_2_S) at the anode and greatly deteriorates the cell performance. The application of symmetrical Solid Oxide Fuel Cells (S-SOFCs) with identical electrode material can easily eliminate or lessen the coking and sulfur poisoning issues by simply changing the gas flow. The traditional cell configuration with three components (anode, electrolyte, cathode) can be replaced by a new approach, where the same electrode material could be used simultaneously as both a cathode and anode to construct a S-SOFC. The symmetrical cells are very promising, due to the reduced amount of cell components, a simplification of the manufacturing process, and by reducing the problems associated with the chemical stability of materials, which, in consequence, will either reduce production costs or ensure a long-term stable operation of SOFCs. Another important benefit related with the application of S-SOFCs will be the ability to address the sulfur poisoning and carbon deposition problems by simply reversing the gas flows when SOFCs are fueled by cheap and available non-hydrogen fuels. In addition, such a symmetrically constructed cell facilitates a reversible operation between fuel cell mode and electrolysis mode. Since the S-SOFC concept was first proposed by a patent of Badding et al. in 2001 [9] and peer-reviewed in articles [10,11] in 2006, it has drawn on a lot of intensive research on the search for novel electrode materials for S-SOFC cells. Furthermore, NASA has developed a novel S-SOFC design with thin YSZ electrolytes using a novel ceramic fabrication technique, generating a good performance (~900 mw cm^−2^) at 850 °C [12]. Recently, the very good electrochemical performance of S-SOFCs with redox-stable electrode materials has been reported [13,14]. However, excellent power outputs exceeding 1000 mWcm^−2^ of S-SOFCs are recorded, rather, at high temperature ranges (around 800 °C) [15,16,17,18,19]. To lower the operating temperature of S-SOFCs while still maintaining a high power output, electrodes with highly active and stable materials are required.

The application of redox-stable double perovskites as electrode materials in S-SOFCs seems to be particularly of interest [14,15,16]. One group of the most interesting electrode materials for SOFCs is the B-site Mo-containing perovskite. The Mo-containing anode materials possess very promising SOFC performance in hydrogen and methane fuels [4,20,21,22]. In particularly, Fe- and Mo-containing Sr_2−x_Ba_x_MMoO_6_ (M = Mg, Mn, Fe, Co, Ni) double perovskite-type oxides, with B-site rock salt-type ordering, are of great interest [23,24,25,26,27]. The present redox pair of Fe^2+^/Fe^3+^ and Mo^6+^/Mo^5+^ in Sr_2_(Fe,Mo)O_6−δ_-type perovskites can provide not only enhanced conductivity [24,25], but also excellent redox stability both in reducing and oxidizing the atmosphere [28,29,30]. In general, the chemical composition consideration in Sr_2_(Fe,Mo)O_6−δ_-type double perovskites is guarded by several criteria. The big difference in the oxidation state of cations between Mo^6+^ or Mo^5+^ (in reducing conditions) and bigger M (3*d* metals, Mg) metals having typically +2 or +3 ensures an appearance of a double perovskite structure with the B-site cations-ordering [4,31]. Moreover, such a mixed valence configuration (Mo^6+^/Mo^5+^ and M^2+^/M^3+^) favors an effective charge transport and also facilitates the creation of oxygen vacancies in materials. Sr_2_(Fe,Mo)O_6−δ_ double perovskite presents metallic conductivity properties while exceeding 1000 S cm^−1^ conductivity in 5 vol.% H_2_/Ar, while unfortunately, the material is not stable in an oxidizing condition [24]. SrFe_0.75_Mo_0.25_O_3−δ_ [28,29], SrFe_0.5_Mn_0.25_Mo_0.25_O_3−δ_ [28], and Sr_1−x_Ba_x_Fe_0.75_W_0.25_O_3−δ_ [32] perovskites show good redox stability and have been evaluated as novel anode materials for S-SOFCs fueled by methane or CO. However, Sr_2_Fe_1.5_Mo_0.5_O_6−δ_ is sensitive to water, and the reaction with water at low temperatures is a potential shortcoming of this material for application in SOFCs [33,34]. The double perovskite (PrBa)_0.95_(Fe_0.9_Mo_0.1_)_2_O_5+δ_ shows very good conductivity of 217 S cm^−1^ in air and 59.2 S cm^−1^ in 5% H_2_ at 800 °C [18]. In addition, the electrode is structurally stable in various fuels, suggesting that the cell can be operated in flexible gas conditions [17]. Interestingly, SOFC cells with (PrBa)_0.95_(Fe_0.9_Mo_0.1_)_2_O_5+δ_ anodes also show outstanding performance in H_2_S-containing fuel (1.18 Wcm^−2^ @800 °C in H_2_+100ppmH_2_S), which indicates this material also has excellent tolerance to sulfur poisoning [18]. The copper-doped Sr_2_Fe_1.5_Mo_0.3_Cu_0.2_O_6−δ_ oxide with enhanced reaction activity and durability was studied for the electrochemical oxidation of H_2_ and reduction of CO_2_, showing a peak SOFC power output of 1.51 W cm^−2^ in hydrogen and a current density of 1.94 A cm^−2^ at 1.4 V in the reduction of CO_2_ to CO [35]. Ti-doped Sr_2_Fe_1.4−x_Ti_x_Mo_0.6_O_6−δ_ double perovskites with an improved structural stability ascribed to the strong Ti-O bond were studied as novel anode materials for SOFCs, with Sr_2_Fe_1.3_Ti_0.1_Mo_0.6_O_6−δ_ anode-based cells delivering a very good power density exceeding 0.64 W cm2 at 900 °C in humidified H_2_ [36]. Sr_2_TiNi_0.5_Mo_0.5_O_6−δ_ was also proposed as a potential oxide anode for SOFCs [37]. Sr_2_FeMo_2/3_Mg_1/3_O_6−δ_ double perovskite due to the presence of Fe_B_-O-Fe_B′_ bonds promoting the easy formation and fast migration of oxygen vacancies in the lattice was proposed as a promising anode material for SOFCs with a good tolerance to carbon deposition and sulfur poisoning [38]. However, the very low electrical conductivity in air (4–5 S cm^−1^ in air 600–800 °C) makes the application of such a compound as a cathode material almost impossible [38]. The effects of Co and Mo doping on the properties of the SrFe_0.45_Co_0.45_Mo_0.1_O_3−δ_ perovskite as an air electrode in reversible solid oxide cells have been investigated [39]. The small bond strength of Co-O contributes to the high mobility of both electron holes and oxide ions, while leading to a high thermal expansion coefficient for the Co-doped composition [39]. Sr_2_Mg_1−x_Co_x_MoO_6−δ_ compounds were proposed as anode materials for SOFCs, and the Co-substitution positively affects the sinterability and ionic conductivity, thus decreasing the anode polarization resistance [40]. However, the weak bond of Co-O causes the stability problem due to the reduction in cobalt to metallic Co in anode conditions. In general, the good transport properties of Fe- and Mo-containing double perovskites can be very promising electrode material candidates for S-SOFCs [18,19,28,29,41].

In this work, magnesium-doped Sr_2_Fe_1.2_Mg_0.2_Mo_0.6_O_6−δ_ and Sr_2_Fe_0.9_Mg_0.4_Mo_0.7_O_6−δ_ double perovskites have been evaluated as potential redox-stable electrode materials for S-SOFCs. The chemical composition of Sr_2_Fe_1.2_Mg_0.2_Mo_0.6_O_6−δ_ and Sr_2_Fe_0.9_Mg_0.4_Mo_0.7_O_6−δ_ has been selected in order to provide good redox stability and electrical conductivity of materials in reducing and oxidizing conditions. The magnesium doping in the proposed compounds can potentially enhance the redox stability, and the content of Mo contributes to the good electrical conductivity [24,28,36,38]. In this study, the physicochemical properties, including the phase composition, crystal structure and its evolution on temperature, redox stability, thermal expansion properties in oxidizing and reducing conditions, oxygen nonstoichiometry and transport properties, as well as chemical stability with electrolytes have been systematically studied.

## 2. Experimental

Sr_2_Fe_1.2_Mg_0.2_Mo_0.6_O_6−δ_ and Sr_2_Fe_0.9_Mg_0.4_Mo_0.7_O_6−δ_ materials were synthesized by the high-temperature solid state reaction method, with calculated stoichiometric amounts of SrCO_3_, Fe_2_O_3_, MgO, and MoO_3_ (all with ≥99.9% purity) compounds as initial chemicals. All needed chemicals were milled in a high-efficiency Spex Sample-Prep 8000 M planetary ball mill, and afterwards, the well-milled powders were pressed into pellets and sintered at 1200 °C for 10 h in air. The crystal structure of the obtained samples was characterized by XRD measurements using a Panalytical Empyrean diffractometer in the 10–110 deg range with CuKα radiation. The redox stability of the studied materials was investigated by the reduction in compounds in 5 vol.% H_2_/Argon at 1000 °C for 10 h. The in situ oxidation of the reduced Sr_2_Fe_1.2_Mg_0.2_Mo_0.6_O_6−δ_ and Sr_2_Fe_0.9_Mg_0.4_Mo_0.7_O_6−δ_ compounds in air was studied by the high-temperature XRD (HT-XRD) measurements performed on a Panalytical Empyrean apparatus equipped with an Anton Paar HTK 1200N oven-chamber. The collected XRD data were refined with the Rietveld method applying the GSAS/EXPGUI software [42,43].

The in situ reduction and oxidation of the investigated sinters were conducted with the thermal expansion studies in 5 vol.% H_2_/Argon and air up to 900 °C on a Linseis L75 Platinum Series dilatometer. Thermogravimetric (TG) analysis was conducted on the TA Instruments Q5000IR apparatus from room temperature (RT) up to 900 °C, with a heating rate of 2°·min^−1^ in different atmospheres (air and argon). The buoyancy effect was also appropriately considered. The oxygen nonstoichiometry δ of Sr_2_Fe_1.2_Mg_0.2_Mo_0.6_O_6−δ_ and Sr_2_Fe_0.9_Mg_0.4_Mo_0.7_O_6−δ_ oxides was determined by the iodometric titration method at RT, and the experimental details can be found in [44]. The titration measurement was performed on an EM40-BNC Mettler Toledo titrator furnished with a platinum electrode. The oxygen nonstoichiometry δ of materials was calculated by using the average values from three independent titration experiments. The total electrical conductivity of the studied compounds was measured up to 900 °C in air and 5 vol.% H_2_/Ar by a four-probe DC method for measuring electrical conductivity (σ). Experiments were performed on dense cuboid shape samples (approx. 3 mm × 4 mm × 10 mm). The porosity effect of the sinters was taken into consideration, with an appropriate correction taken from Bruggeman’s effective medium approximation [45]. The oxygen diffusion coefficient *D* and surface exchange coefficient *k* of Sr_2_Fe_1.2_Mg_0.2_Mo_0.6_O_6−δ_ oxide were also evaluated by the mass relaxation technique using TA Instruments Q5000 IR apparatus on thin-sheet shape sinter [46,47]. The mass relaxation data were collected during the rapid change of oxygen partial pressure between 0.21 atm and 0.01 atm. The calculation of the chemical diffusion coefficient *D* and surface exchange constant *k* was performed in a custom-made Matlab code, based on mathematical solutions provided by Crank [48]. The chemical stability and compatibility studies of Sr_2_Fe_1.2_Mg_0.2_Mo_0.6_O_6−δ_ and Sr_2_Fe_0.9_Mg_0.4_Mo_0.7_O_6−δ_ oxides towards typical solid electrolytes CGO20 (Ce_0.8_Gd_0.2_O_1.9_) and LSGM (La_0.8_Sr_0.2_Ga_0.8_Mg_0.2_O_3−d_) were examined by analyzing the XRD data collected for the respective oxide and the electrolyte powder mixtures (50:50 wt.%), which were annealed at 1200 °C for 4 h in air.

## 3. Results and Discussion

### 3.1. Crystal Structure and the Redox Stability

**Figure 1:**
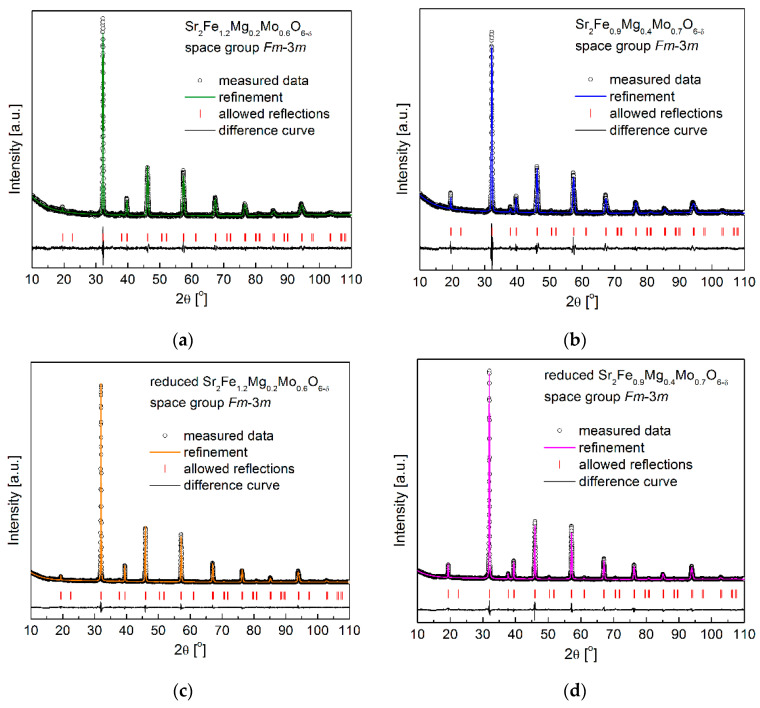
XRD patterns recorded for (**a**) as-synthesized Sr_2_Fe_1.2_Mg_0.2_Mo_0.6_O_6−δ_ and (**b**) Sr_2_Fe_0.9_Mg_0.4_Mo_0.7_O_6−δ_, (**c**) reduced Sr_2_Fe_1.2_Mg_0.2_Mo_0.6_O_6−δ_ and (**d**) Sr_2_Fe_0.9_Mg_0.4_Mo_0.7_O_6−δ_.

Figure 1a,b show the XRD patterns with the Rietveld refinement of the as-synthesized Sr_2_Fe_1.2_Mg_0.2_Mo_0.6_O_6−δ_ and Sr_2_Fe_0.9_Mg_0.4_Mo_0.7_O_6−δ_ compounds, which were sintered for 10 h at 1200 °C in air. Both samples exhibit a B-site double perovskite structure with an *Fm*-3*m* space group, and no other impurities can be observed in the detection limit. A similar double perovskite structure (*Fm*-3*m* space group) has also been reported for Sr_2_FeMo_2/3_Mg_1/3_O_6−δ_ [38], Ti-doped Sr_2_Fe_1.4−x_Ti_x_Mo_0.6_O_6−δ_ (x = 0 and 0.1) [36], and Sr_2_Fe_1.5_Mo_0.5_O_6−δ_ oxides [29]. Interestingly, Sr_2_FeMoO_6−δ_ [24] and Sr_2_MgMoO_6−δ_ [49] oxides possess lower symmetry with the *I*4/*m* and *I*-1 space groups, respectively. The structural parameters based on the Rietveld refinement results of the studied materials are presented in Table 1. The increased content of magnesium doping in materials leads to the increase in unit cell parameter *a* from 7.8604 Å (for Sr_2_Fe_1.2_Mg_0.2_Mo_0.6_O_6−δ_) to 7.8709Å (for Sr_2_Fe_0.9_Mg_0.4_Mo_0.7_O_6−δ_), which is due to the large ionic radius of Mg^2+^ (*r*_Mg2+_ = 0.72 Å). As the Sr_2_Fe_1.2_Mg_0.2_Mo_0.6_O_6−δ_ and Sr_2_Fe_0.9_Mg_0.4_Mo_0.7_O_6−δ_ double perovskites were synthesized in the air, the presence of Fe^3+^/Fe^4+^ (*r*_Fe3+_ = 0.645 Å and *r*_Fe4+_ = 0.585 Å) and Mo^6+^ (*r*_Mo6+_ = 0.59 Å) should be dominant. Therefore, the doping of the much larger Mg^2+^ in the studied materials should increase the unit cell parameters as observed in Table 1. The geometric tolerance factor *t_g_* was also calculated with the equation $t_{g}=\frac{\left[A-O\right]}{\sqrt{2}[B-O]}$, where [A − O] and [B − O] are the appropriate geometric averages of the refined interatomic distances of Sr-O and M-O (M: Fe, Mg and Mo), respectively [24]. The tolerance factors *t_g_* calculated for Sr_2_Fe_1.2_Mg_0.2_Mo_0.6_O_6−δ_ and Sr_2_Fe_0.9_Mg_0.4_Mo_0.7_O_6−δ_ oxides are 1.00 and 0.963, respectively.

**Figure 2:**
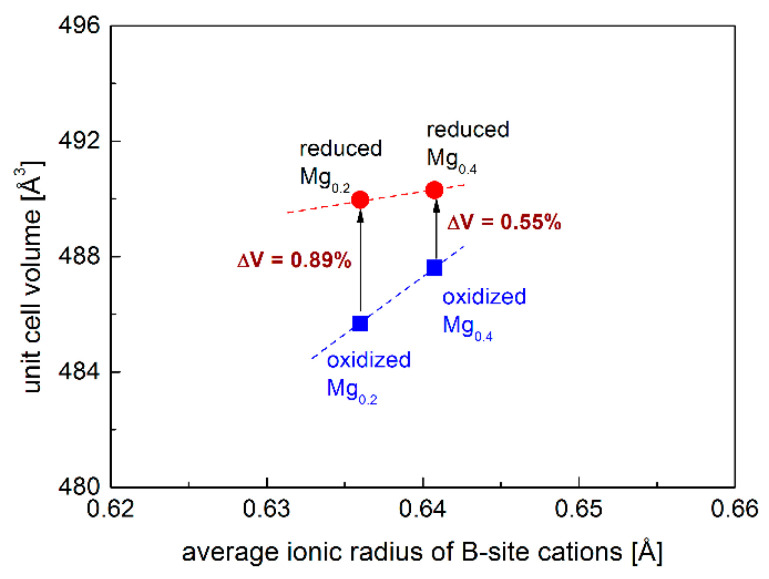
Normalized unit cell volume of reduced and oxidized Sr_2_Fe_1.2_Mg_0.2_Mo_0.6_O_6−δ_ (Mg_0.2_), Sr_2_Fe_0.9_Mg_0.4_Mo_0.7_O_6−δ_ (Mg_0.4_) as a function of B-site cations’ ionic radius. Error values are smaller than the visible points. The average ionic radius of B-site cations was calculated only using the ionic radius of Fe^3+^ and Mo^6+^ cations for a direct comparison of the relative volume change of reduced and oxidized oxides.

As the electrode materials for S-SOFCs working both in the oxidization and reduction of the atmosphere, the redox stability of investigated samples is very crucial. Sr_2_Fe_1.2_Mg_0.2_Mo_0.6_O_6−δ_ and Sr_2_Fe_0.9_Mg_0.4_Mo_0.7_O_6−δ_ were reduced in 5 vol.% H_2_/Argon at 1000 °C for 10 h, and the XRD patterns are presented in Figure 1c,d, which show the redox stability of Mg-doped materials. The reduced samples present the same crystal structure (*Fm*-3*m*), while the unit cell parameters *a* slightly increase (see Table 1). This is related to the reduction of Fe^3+^/Fe^4+^ to Fe^3+^/Fe^2+^ and Mo^6+^ to Mo^5+^/Mo^4+^ [28]. Interestingly, the relative unit cell volume change ∆*V* between the reduced and oxidized samples is very small, and the increased Mg-doping content decreases the volume change ∆*V* (Figure 2), which is associated with the redox-stable Mg^2+^ and the decreased content of Fe with changeable oxidation states in the sample. In particular, the Sr_2_Fe_0.9_Mg_0.4_Mo_0.7_O_6−δ_ compound possesses a very small volume change, ∆*V* = 0.55%, which favors the application of such a material both in reducing and oxidizing conditions [46]. For comparison, the relative volume change between the reduced and oxidized Sr_2_Fe_1.5_Mo_0.5_O_6−δ_ oxide is much larger and reaches 1.18% [28].

**Figure 3:**
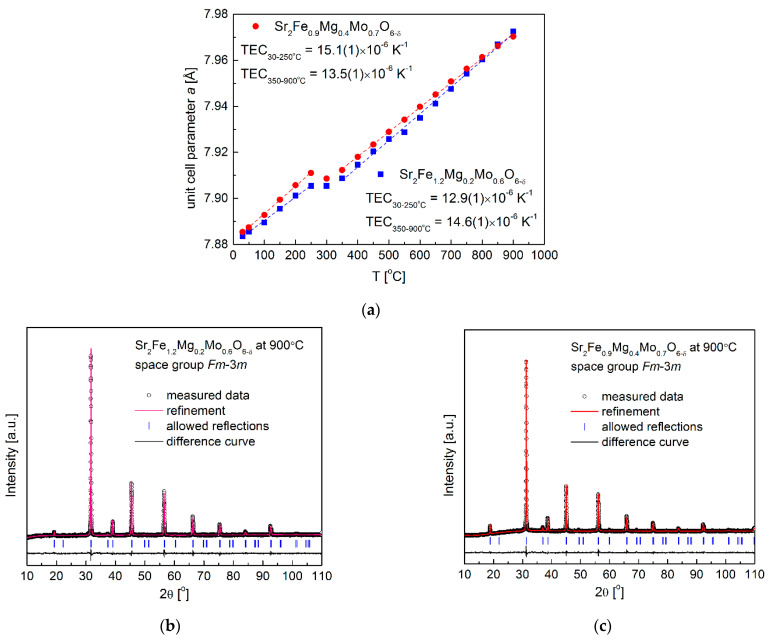
(**a**) In situ XRD measurements of oxidizing reduced Sr_2_Fe_1.2_Mg_0.2_Mo_0.6_O_6−δ_ and Sr_2_Fe_0.9_Mg_0.4_Mo_0.7_O_6−δ_ oxides in air from 30–900 °C, (**b**) XRD patterns recorded for Sr_2_Fe_1.2_Mg_0.2_Mo_0.6_O_6−δ_ and (**c**) Sr_2_Fe_0.9_Mg_0.4_Mo_0.7_O_6−δ_ samples at 900 °C in air.

For further studies of the redox stability of electrode materials, the reduced Sr_2_Fe_1.2_Mg_0.2_Mo_0.6_O_6−δ_ and Sr_2_Fe_0.9_Mg_0.4_Mo_0.7_O_6−δ_ samples were in situ oxidized in air utilizing HT-XRD measurements (Figure 3a–c). The lattice parameters of samples at different temperatures can be calculated by the Rietveld refinement of collected HT-XRD data, and the obtained unit cell parameter variations of the investigated compounds are depicted in Figure 3a. Firstly, the unit cell parameter *a* of both the two samples linearly increases with the temperature to 250 °C, showing the thermal expansion of reduced materials in this low temperature range. The significant (non-linear) change in the lattice parameter occurs at around 300 °C, indicating the oxidation of the reduced samples. The oxidation of the reduced samples causes the decrease in the unit cell parameter *a*, which is due to the oxidation of Fe^2+^/Fe^3+^ and Mo^4+^/Mo^5+^ to high oxidation states (Fe^3+^/Fe^4+^ and Mo^6+^). The already oxidized materials show linear thermal expansion above 350 °C. The thermal expansion coefficients (TEC) of samples shown in the inset can be derived with the fitted linear slope. In general, the TECs obtained from the HT-XRD measurements for both the two materials are relatively low and in the range of 12.9–15.1 × 10^−6^ K^−1^. Those values are comparable with the TEC values of typical electrolytes [24], which indicates the possible good mechanical cohesion of electrode materials with electrolytes. For both the two materials, no phase transition has been observed, and the crystal structure with the *Fm*-3*m* space group is stabilized up to 900 °C (Figure 3b,c).

### 3.2. Thermal Expansion Properties and Oxygen Nonstoichiometry

**Figure 4:**
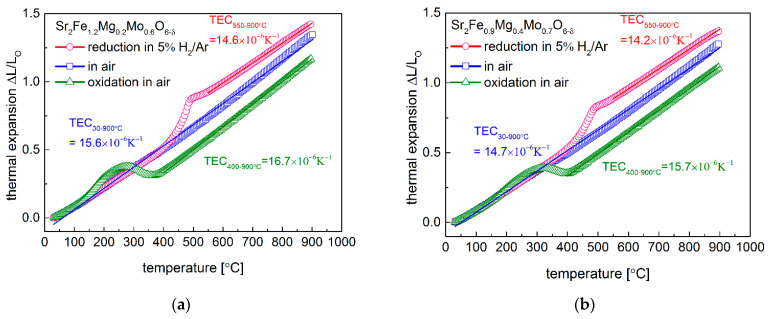
Thermal expansion behavior of (**a**) Sr_2_Fe_1.2_Mg_0.2_Mo_0.6_O_6−δ_ and (**b**) Sr_2_Fe_0.9_Mg_0.4_Mo_0.7_O_6−δ_ materials in air, reduction in 5 vol.% H_2_/argon, and oxidization in air of the reduced oxides.

**Figure 5:**
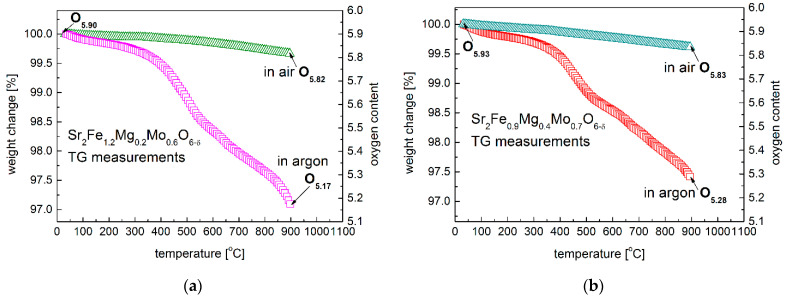
Oxygen content change of (**a**) Sr_2_Fe_1.2_Mg_0.2_Mo_0.6_O_6−δ_ and (**b**) Sr_2_Fe_0.9_Mg_0.4_Mo_0.7_O_6−δ_ oxides in air and argon, respectively.

The in situ reduction and oxidation of Sr_2_Fe_1.2_Mg_0.2_Mo_0.6_O_6−δ_ and Sr_2_Fe_0.9_Mg_0.4_Mo_0.7_O_6−δ_ oxides were examined by applying dilatometer measurements (Figure 4a,b). In air, Sr_2_Fe_1.2_Mg_0.2_Mo_0.6_O_6−δ_ sample (TEC = 15.6 × 10^−6^ K^−1^) shows slightly higher TEC values than Sr_2_Fe_0.9_Mg_0.4_Mo_0.7_O_6−δ_ (TEC = 14.7 × 10^−6^ K^−1^), which indicates that the magnesium doping suppresses the thermal expansion of materials. In addition, the lower content of oxygen vacancies in Sr_2_Fe_0.9_Mg_0.4_Mo_0.7_O_6−δ_ also contributes to a smaller TEC than that of Sr_2_Fe_1.2_Mg_0.2_Mo_0.6_O_6−δ_. The in situ reduction in Sr_2_Fe_1.2_Mg_0.2_Mo_0.6_O_6−δ_ and Sr_2_Fe_0.9_Mg_0.4_Mo_0.7_O_6−δ_ samples were conducted by dilatometer studies in 5 vol.% H_2_/Ar, and a linear thermal expansion of both materials as a function of temperature was documented up to around 400 °C. The continuous reduction of materials leads to a considerable expansion of the samples, which is associated with the reduction of Fe^3+^/Fe^4+^ and Mo^6+^ to low oxygen states, and a similar reduction behavior was also recorded in the TG measurements (Figure 5a,b). The significant reduction of Fe and Mo was up to 550 °C, while the further increase in the temperature contributed to a linear thermal expansion of the reduced samples. The reduced samples in 5 vol.% H_2_/Ar possess relatively low thermal expansion values above 550 °C, with 14.6 × 10^−6^ K^−1^ for Sr_2_Fe_1.2_Mg_0.2_Mo_0.6_O_6−δ_ and 14.2 × 10^−6^ K^−1^ for Sr_2_Fe_0.9_Mg_0.4_Mo_0.7_O_6−δ_. Moreover, the reduced sinters were in situ oxidized in air with dilatometer measurements (Figure 4a,b). In the case of reduced Sr_2_Fe_1.2_Mg_0.2_Mo_0.6_O_6−δ_, the linear thermal expansion is below 150 °C, while for Sr_2_Fe_0.9_Mg_0.4_Mo_0.7_O_6−δ_, the reduced sample linearly expands to around 200 °C, corresponding well with the linear expansion of the lattice parameters measured in the HT-XRD studies (Figure 3a). The continuous oxidation of reduced sinters leads to an interval shrinkage for the samples, which is due to the decrease in the unit cell parameters recorded in Figure 3a, caused by the oxidation of Fe^2+^/Fe^3+^ and Mo^4+^/Mo^5+^ to Fe^3+^/Fe^4+^ and Mo^6+^. Above 400 °C, the samples are fully oxidized and show further linear expansion properties. The magnesium doping in Sr_2_Fe_0.9_Mg_0.4_Mo_0.7_O_6−δ_ (TEC = 15.7 × 10^−6^ K^−1^) decreases the TEC value in the reducing condition when compared with Sr_2_Fe_1.2_Mg_0.2_Mo_0.6_O_6−δ_ sample. In general, Sr_2_Fe_0.9_Mg_0.4_Mo_0.7_O_6−δ_ oxide possesses smaller TEC values than Sr_2_Fe_1.2_Mg_0.2_Mo_0.6_O_6−δ_ (see Table 2). Therefore, Mg doping in materials suppresses the thermal expansion of samples in the oxidization and reduction of atmospheres. Importantly, for both the Sr_2_Fe_1.2_Mg_0.2_Mo_0.6_O_6−δ_ and Sr_2_Fe_0.9_Mg_0.4_Mo_0.7_O_6−δ_ compounds, no visible cracks were detected after the in situ reduction and oxidation studies by the dilatometer measurements, which proved the excellent redox stability of the investigated materials. The TEC values of the materials collected from the dilatometer studies and HT-XRD measurements in the oxidizing and reducing conditions are in the range of 12.9–15.7 × 10^−6^ K^−1^. The TEC values of Sr_2_Fe_1.2_Mg_0.2_Mo_0.6_O_6−δ_ and Sr_2_Fe_0.9_Mg_0.4_Mo_0.7_O_6−δ_ are relatively small in the oxidizing and reducing conditions (see Table 2), and those values are very close to the TECs of typical electrolytes, such as: La_0.9_Sr_0.1_Ga_0.8_Mg_0.2_O_3−δ_—12.17 × 10^−6^ K^−1^, Zr_0.85_Y_0.15_O_2−δ_—10.8 × 10^−6^ K^−1^ and Ce_0.8_Gd_0.2_O_2−δ_—12.5 × 10^−6^ K^−1^ [24]. Therefore, in the case of the application of redox-stable Sr_2_Fe_1.2_Mg_0.2_Mo_0.6_O_6−δ_ and Sr_2_Fe_0.9_Mg_0.4_Mo_0.7_O_6−δ_ double perovskites as electrode materials for S-SOFCs, the delamination problem is alleviated, possibly providing a stable cell performance.

The oxygen content of Sr_2_Fe_1.2_Mg_0.2_Mo_0.6_O_6−δ_ and Sr_2_Fe_0.9_Mg_0.4_Mo_0.7_O_6−δ_ at room temperature was determined by iodometric titration. Sr_2_Fe_1.2_Mg_0.2_Mo_0.6_O_5.90_ and Sr_2_Fe_0.9_Mg_0.4_Mo_0.7_O_5.93_ are obtained at room temperature, which shows the presence of the same average iron oxidation state, with +3.17 in both materials. This indicates the considerable dominant existence of Fe^3+^ (83.3%) and a small amount of Fe^4+^ (16.7%) present in both Sr_2_Fe_1.2_Mg_0.2_Mo_0.6_O_5.90_ and Sr_2_Fe_0.9_Mg_0.4_Mo_0.7_O_5.93_ perovskites. The oxygen content change of Sr_2_Fe_1.2_Mg_0.2_Mo_0.6_O_6−δ_ and Sr_2_Fe_0.9_Mg_0.4_Mo_0.7_O_6−δ_ oxides was measured as a function of temperature up to 900 °C by TG studies in air and in argon, respectively (Figure 5a,b). The measured oxygen content of materials is presented in Table 3. Both the two samples show a similar content of oxygen nonstoichiometry at 900 °C in air, with δ = 0.18 for Sr_2_Fe_1.2_Mg_0.2_Mo_0.6_O_6−δ_ and δ = 0.17 for Sr_2_Fe_0.9_Mg_0.4_Mo_0.7_O_6−δ_. The additional oxygen vacancies in the high-temperature range are created in the materials, according to the reaction: $O_{O}^{X}↔1/2O_{2}+V_{O}^{••}+2e^{-}$. In the argon atmosphere, a substantial oxygen content change has been recorded for both the two materials. The initial start of a considerable oxygen content drop is at around 350 °C. Since the reduction energies of Fe^3+^/Fe^2+^ are much lower than that of Mo^6+^/Mo^5+^ [4,28], the first reduction in the samples can be ascribed to the reduction in iron cations to Fe^2+^. The second step change (above 550 °C) can be related with the reduction in Mo^6+^ to Mo^5+^/Mo^4+^ and the further increase in the oxygen vacancies concentration [28]. The oxygen contents of Sr_2_Fe_1.2_Mg_0.2_Mo_0.6_O_6−δ_ and Sr_2_Fe_0.9_Mg_0.4_Mo_0.7_O_6−δ_ samples in argon are 5.17 and 5.28, respectively. Therefore, the relative oxygen nonstoichiometry changes ∆δ of Sr_2_Fe_1.2_Mg_0.2_Mo_0.6_O_6−δ_ and Sr_2_Fe_0.9_Mg_0.4_Mo_0.7_O_6−δ_ samples in argon up to 900 °C are 0.73 and 0.65, respectively. The higher content of redox couples of Fe^3+^/Fe^2+^ and Mo^6+^/Mo^5+^ in less Mg-doped Sr_2_Fe_1.2_Mg_0.2_Mo_0.6_O_6−δ_ allows ro4 a generation of more oxygen vacancies than in Sr_2_Fe_0.9_Mg_0.4_Mo_0.7_O_6−δ_, which indicates that the increased content of magnesium in materials does not favor the creation of oxygen vacancies.

### 3.3. The Transport Properties

**Figure 6:**
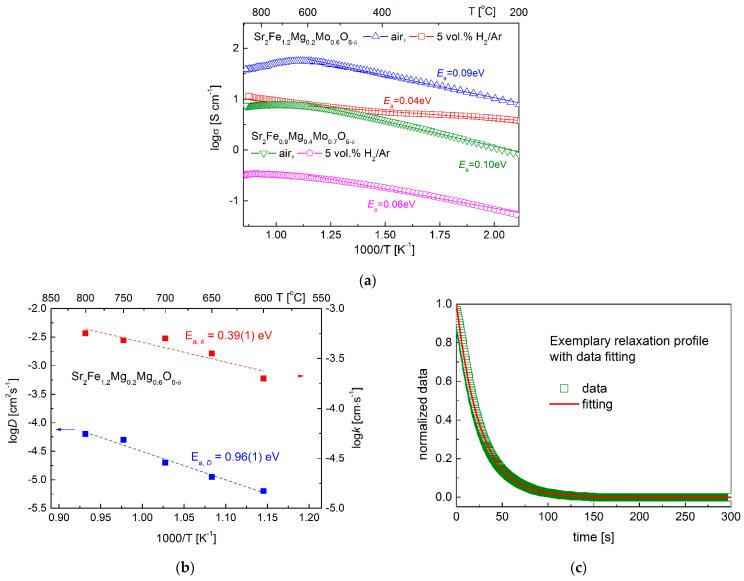
(**a**) Electrical conductivity of Sr_2_Fe_1.2_Mg_0.2_Mo_0.6_O_6−δ_ and Sr_2_Fe_0.9_Mg_0.4_Mo_0.7_O_6−δ_ materials in air and 5 vol.% H_2_/Ar, (**b**) diffusion coefficients *D* and *k* measured as a function of temperature for Sr_2_Fe_1.2_Mg_0.2_Mo_0.6_O_6−δ_ sample, and (**c**) an exemplary normalized relaxation profile with fitting.

The electrical conductivity data gathered between 200–900 °C for Sr_2_Fe_1.2_Mg_0.2_Mo_0.6_O_6−δ_ and Sr_2_Fe_0.9_Mg_0.4_Mo_0.7_O_6−δ_ samples show the increased doping content of magnesium decreases the total conductivity and increases the activation energy in air and 5 vol. H_2_/Ar (Figure 6a). In materials, the electrons are transmitted via the Fe^2+^/Fe^3+^-O^2—^Fe^3+^/Fe^4+^ network. The Mg doping in the samples reduces the content of iron, thus resulting in the decrease in electrical conductivities. For both the two samples, the conductivity measured in air shows a maximum value with temperature: it increases initially and then drops. Samples exhibit a linear relationship in the low-temperature range (<600 °C), indicating small polaron conduction behavior. Sr_2_Fe_1.2_Mg_0.2_Mo_0.6_O_6−δ_ (*E*_a_ = 0.09 eV) possesses a slightly smaller activation energy than that of Sr_2_Fe_0.9_Mg_0.4_Mo_0.7_O_6−δ_ (*E*_a_ = 0.10 eV). In the case of Sr_2_Fe_1.2_Mg_0.2_Mo_0.6_O_6−δ_, the maximum conductivity of 56.2 S·cm^−1^ was recorded at around 600 °C, while for Sr_2_Fe_0.9_Mg_0.4_Mo_0.7_O_6−δ_, the peak value with 8.0 S·cm^−1^ was measured at 700 °C. The conductivity decreases with the further increase in the temperature, which can be associated with the release of lattice oxygen ($O_{O}^{X}↔1/2O_{2}+V_{O}^{••}+2e^{-}$) at high temperatures breaking the Fe^3+^-O^2-^-Fe^4+^ network, resulting in the decrease in electrical conductivities. Similar phenomena have been recorded in other SrFeO_3_-based materials [28,41]. In the reducing atmosphere (5 vol.% H_2_/Ar), both Sr_2_Fe_1.2_Mg_0.2_Mo_0.6_O_6−δ_ and Sr_2_Fe_0.9_Mg_0.4_Mo_0.7_O_6−δ_ compounds show much lower conductivity. The Sr_2_Fe_1.2_Mg_0.2_Mo_0.6_O_6−δ_ sample possesses a relatively satisfactory conductivity value (10.3 S·cm^−1^ at 800 °C) with a very small activation energy *E*_a_ = 0.04 eV. The Sr_2_Fe_1.2_Mg_0.2_Mo_0.6_O_6−δ_ material shows much higher conductivity in air and comparable values in the reducing condition with Sr_2_FeMo_2/3_Mg_1/3_O_6−δ_ oxide (4–5 S·cm^−1^ in air, and 9–13 S·cm^−1^ in H_2_ at 600–800 °C) [38]. In addition, the measured electrical conductivity of Sr_2_Fe_1.2_Mg_0.2_Mo_0.6_O_6−δ_ is much higher than the σ value of Sr_2_MgMoO_6−δ_ [49], Sr_2_Fe_1.5_Mo_0.3_Cu_0.2_O_6−δ_ [35], Sr_2−x_Ba_x_MgMoO_6−δ_, and Sr_2−x_Ba_x_MnMoO_6−δ_ [24,25]. In addition, the chemical diffusion coefficient *D* and surface exchange constant *k* of Sr_2_Fe_1.2_Mg_0.2_Mo_0.6_O_6−δ_ have been determined by the mass relaxation technique (Figure 6b,c). The *D* coefficient is in the range of 6.3 × 10^−6^ to 6.3 × 10^−5^ cm^2^ s^−1^ at 600–800 °C, with the activation energy *E*_a,D_ = 0.96 eV. The *k* constant is within the scope of 2.0 × 10^−4^–5.6 × 10^−4^ cm s^−1^, with the activation energy *E*_a,k_ = 0.396 eV. The measured chemical diffusion coefficients *D* are comparable with the *D* recorded for Sr_2_TiNi_0.5_Mo_0.5_O_6−δ_ [37] and Sr_2_Fe_1.4_Mn_0.1_Mo_0.5_O_6−δ_ [50] materials, while the *k* values of Sr_2_Fe_1.2_Mg_0.2_Mo_0.6_O_6−δ_ are slightly smaller than *k* of Sr_2_Fe_1.4_Mn_0.1_Mo_0.5_O_6−δ_ [50]. The relatively high diffusion coefficients *D* and *k* measured for Sr_2_Fe_1.2_Mg_0.2_Mo_0.6_O_6−δ_ oxide indicate the good ionic transport in such a material. 

The ionic conductivity σ_i_ of Sr_2_Fe_1.2_Mg_0.2_Mo_0.6_O_6−δ_ oxide was also evaluated using the Nernst–Einstein equation: $\sigma_{i}=\frac{c_{O}\cdot q^{2}D_{s}}{RT}=\frac{2c_{O}\cdot q^{2}D\cdot dlnc_{O}}{RT\cdot dlnpO_{2}}$, where: *c_O_*—concentration of oxygen, *q*—charge of mole of oxygen anions, *D_s_*—self-diffusion coefficient, *R*—gas constant, *T*—temperature [51]. The ionic conductivity *σ_i_* of Sr_2_Fe_1.2_Mg_0.2_Mo_0.6_O_6−δ_ oxide was estimated to be about 0.005 S·cm^−1^ at 800 °C. For the calculation, the high-temperature XRD-based density of the sample recorded at 800 °C was used, together with the determined oxygen content, allowing the obtaining of the *c_O_* value. In the calculation, all oxygen anions were considered as participating in the ionic transport, which may be a factor generating the biggest error for the performed evaluation. Despite the mentioned error source, the evaluated ionic conductivity *σ_i_* of Sr_2_Fe_1.2_Mg_0.2_Mo_0.6_O_6−δ_ is reasonable and comparable with the measured *σ_i_* values for Sr_2_Mg_1−x_Co_x_MoO_6−δ_ (x = 0, 0.3 and 0.7) samples at 800 °C [40]. Therefore, compared with the most crucial physicochemical proprieties of Sr_2_(Fe,Mo)O_6−δ_-type materials presented in the Table 4, Sr_2_Fe_1.2_Mg_0.2_Mo_0.6_O_6−δ_ oxide can potentially be a good electrode material candidate for S-SOFCs.

### 3.4. Chemical Stability and Compatibility of Electrode Materials with Electrolytes

**Figure 7:**
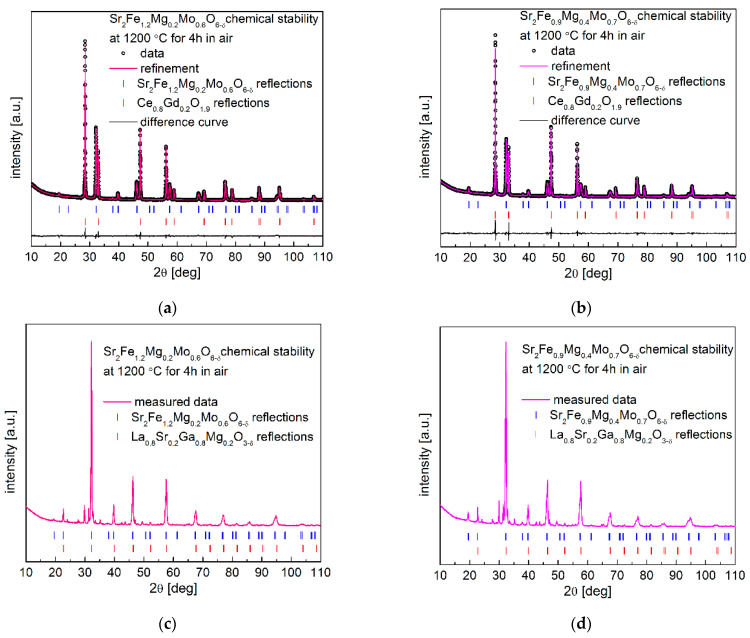
XRD patterns recorded for (**a**) Sr_2_Fe_1.2_Mg_0.2_Mo_0.6_O_6−δ_ and (**b**) Sr_2_Fe_0.9_Mg_0.4_Mo_0.7_O_6−δ_ with CGO20 electrolyte, (**c**) Sr_2_Fe_1.2_Mg_0.2_Mo_0.6_O_6−δ_, and (**d**) Sr_2_Fe_0.9_Mg_0.4_Mo_0.7_O_6−δ_ with LSGM electrolyte, after sintering at 1200 °C for 4 h in air.

The chemical stability of electrode materials and their compatibility with the used electrolytes in an operating temperature range are critical for the long and stable performance of SOFCs. Therefore, the chemical stability and compatibility of Sr_2_Fe_1.2_Mg_0.2_Mo_0.6_O_6−δ_ and Sr_2_Fe_0.9_Mg_0.4_Mo_0.7_O_6−δ_ with typical electrolytes Ce_0.8_Gd_0.2_O_1.9_ (CGO20) and La_0.8_Sr_0.2_Ga_0.8_Mg_0.2_O_3−d_ (LSGM) have been investigated at 1200 °C for 4 h in air. The Rietveld-refined XRD patterns of Sr_2_Fe_1.2_Mg_0.2_Mo_0.6_O_6−δ_ and Sr_2_Fe_0.9_Mg_0.4_Mo_0.7_O_6−δ_ with electrolyte (50:50 wt.%) powder mixtures are presented in Figure 7a–d. In Figure 7a,b, the XRD data after the studies of electrode materials with CGO20 show no additional phase, confirming the good chemical stability and compatibility of the electrode materials with the Ce_0.8_Gd_0.2_O_1.9_ electrolyte. Moreover, as can be observed from Table 5, the refined unit cell parameters of Sr_2_Fe_1.2_Mg_0.2_Mo_0.6_O_6−δ_ and Sr_2_Fe_0.9_Mg_0.4_Mo_0.7_O_6−δ_ after the chemical stability and compatibility studies are slightly smaller but very close to the results of as-synthesized materials presented in Table 1. The smaller unit cell parameters recorded after the annealing at 1200 °C for 4 h in air indicates the increased content of present Fe^4+^ (high oxygen state) in the materials. The studies show the possible long stable operation of SOFCs with Sr_2_Fe_1.2_Mg_0.2_Mo_0.6_O_6−δ_ and Sr_2_Fe_0.9_Mg_0.4_Mo_0.7_O_6−δ_ electrode materials towards the typical electrolyte Ce_0.8_Gd_0.2_O_1.9_. However, the studies on Sr_2_Fe_1.2_Mg_0.2_Mo_0.6_O_6−δ_ and Sr_2_Fe_0.9_Mg_0.4_Mo_0.7_O_6−δ_ with La_0.8_Sr_0.2_Ga_0.8_Mg_0.2_O_3−d_ show the incompatibility of electrode materials with LSGM electrolyte, with the appearance of additional impurities observed in Figure 7c,d.

**Figure 8:**
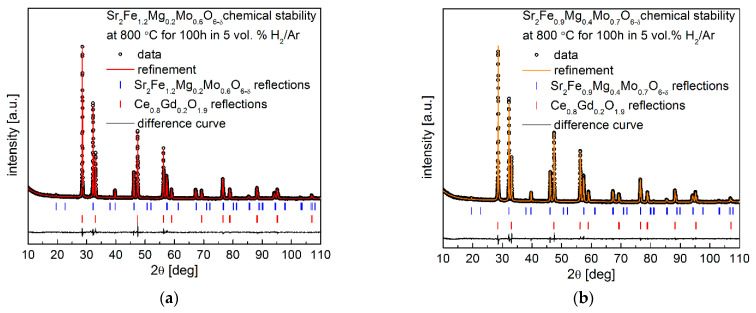
XRD patterns recorded for (**a**) Sr_2_Fe_1.2_Mg_0.2_Mo_0.6_O_6−δ_ and (**b**) Sr_2_Fe_0.9_Mg_0.4_Mo_0.7_O_6−δ_ with the Ce_0.8_Gd_0.2_O_1.9_ electrolyte after annealing in 5 vol.% H_2_/Ar at 800 °C for 100 h.

In addition, the long-term chemical compatibility of the Sr_2_Fe_1.2_Mg_0.2_Mo_0.6_O_6−δ_ and Sr_2_Fe_0.9_Mg_0.4_Mo_0.7_O_6−δ_ electrode materials with Ce_0.8_Gd_0.2_O_1.9_ was investigated in 5 vol.% H_2_ in argon at 800 °C for 100 h. The collected XRD data after such studies shown in Figure 8a,b also confirm the excellent chemical stability and compatibility of the proposed electrode materials with the Ce_0.8_Gd_0.2_O_1.9_ electrolyte. Interestingly, as expected, the unit cell parameters of Sr_2_Fe_1.2_Mg_0.2_Mo_0.6_O_6−δ_ and Sr_2_Fe_0.9_Mg_0.4_Mo_0.7_O_6−δ_ after the long-term reduction at 800 °C (Table 6) are smaller than the values of materials reduced at 1000 °C (See Table 1). This is related to the lower content of reduced iron and molybdenum cations (contributing to smaller unit cell parameters) in materials annealed at 800 °C in 5 vol.% H_2_/Ar. The unit cell parameters of Ce_0.8_Gd_0.2_O_1.9_ presented in Table 6 are very close but a little bit bigger than the values of Ce_0.8_Gd_0.2_O_1.9_ recorded in Table 5, indicating the stability of Ce_0.8_Gd_0.2_O_1.9_ in the presence of studied electrode materials and a slight reduction in Ce_0.8_Gd_0.2_O_1.9_ in the atmosphere of 5 vol.% H_2_/Ar.

## 4. Conclusions

The physicochemical properties including the crystal structure properties, redox stability, thermal expansion properties in oxidizing and reducing conditions, oxygen nonstoichiometry as a function of temperature and transport properties, as well as the chemical compatibility with typical electrolytes have been systematically studied for magnesium-doped Sr_2_Fe_1.2_Mg_0.2_Mo_0.6_O_6−δ_ and Sr_2_Fe_0.9_Mg_0.4_Mo_0.7_O_6−δ_ oxides. Sr_2_Fe_1.2_Mg_0.2_Mo_0.6_O_6−δ_ and Sr_2_Fe_0.9_Mg_0.4_Mo_0.7_O_6−δ_ compounds crystallize in *Fm*-3*m* space group with a double perovskite structure, which is stable in reducing and oxidizing conditions and up to a high-temperature range. The relative volume change between reduced and oxidized materials is very small (especially for Sr_2_Fe_0.9_Mg_0.4_Mo_0.7_O_6−δ_, ∆V = 0.55%), which favors the application of such a material both in reducing and oxidizing conditions. The in situ reduction and oxidation of sinters with dilatometer measurements prove the excellent redox stability of investigated materials in reducing and oxidizing conditions, with TEC values measured very close to the most used solid electrolytes. Mg doping in materials suppresses the thermal expansion of samples in oxidizing and reducing atmospheres. Both materials show a presence of oxygen vacancies, and the oxygen content was determined at room temperature (Sr_2_Fe_1.2_Mg_0.2_Mo_0.6_O_5.90_ and Sr_2_Fe_0.9_Mg_0.4_Mo_0.7_O_5.93_) and recorded up to a high temperature range (up to 900 °C), while the increase in magnesium content in materials decreased the oxygen nonstoichiometry δ. Sr_2_Fe_1.2_Mg_0.2_Mo_0.6_O_6−δ_ perovskite presents relatively good electrical conductivity in air (56.2 S·cm^−1^ at 600 °C) and 5 vol.% H_2_/Ar (10.3 S·cm^−1^ at 800 °C). The measured relatively high chemical diffusion coefficient *D* and surface exchange constant *k* indicates the good ionic conductivity of the material. In addition, the ionic conductivity was also calculated with the value of 0.005 S·cm^−1^ at 800 °C. The stability studies show that both compounds are compatible with Ce_0.8_Gd_0.2_O_1.9_ but react with the La_0.8_Sr_0.2_Ga_0.8_Mg_0.2_O_3−d_ electrolyte. Therefore, the magnesium-doped Sr_2_Fe_1.2_Mg_0.2_Mo_0.6_O_6−δ_ double perovskite with excellent redox stability can be potentially applied as stable electrode materials for symmetrical SOFCs and are of great interest for further investigations.

## Figures and Tables

**Table 1:** Rietveld refinement results for as-synthesized and reduced Sr_2_Fe_1.2_Mg_0.2_Mo_0.6_O_6−δ_ and Sr_2_Fe_0.9_Mg_0.4_Mo_0.7_O_6−δ_ oxides.

Composition	Sr_2_Fe_1.2_Mg_0.2_Mo_0.6_O_6−δ_		Sr_2_Fe_0.9_Mg_0.4_Mo_0.7_O_6−δ_	
T [°C]	as-Synthesized	Reduced at 1000 °C	as-Synthesized	Reduced at 1000 °C
space group	*Fm*-3*m*	*Fm*-3*m*	*Fm*-3*m*	*Fm*-3*m*
*a* [Å]	7.8604(1)	7.8836(1)	7.8709(1)	7.8854(1)
*V* [Å^3^]	485.67(1)	489.97(1)	487.61(1)	490.30(1)
density [g/cm^3^]	5.48	5.43	5.43	5.40
R_p_ (%)	2.13	1.43	2.62	1.77
R_wp_ (%)	2.84	2.02	3.89	2.74

**Table 2:** Thermal expansion coefficients TEC [10^–6^ K^–1^] of Sr_2_Fe_1.2_Mg_0.2_Mo_0.6_O_6–δ_ and Sr_2_Fe_0.9_Mg_0.4_Mo_0.7_O_6–δ_ oxides from dilatometer measurements and high-temperature XRD studies in air.

	HT-XRD (30–250 °C)	HT-XRD (350–900 °C)	Dilatometer (30–900 °C in Air)	Dilatometer (400–900 °C, Oxidation in Air)	Dilatometer (550–900 °C, Reduction in 5% H_2_/Ar)
Sr_2_Fe_1.2_Mg_0.2_Mo_0.6_O_6−δ_	12.9	14.6	15.6	16.7	14.6
Sr_2_Fe_0.9_Mg_0.4_Mo_0.7_O_6−δ_	15.1	13.5	14.7	15.7	14.2

**Table 3:** Oxygen content of Sr_2_Fe_1.2_Mg_0.2_Mo_0.6_O_6−δ_ and Sr_2_Fe_0.9_Mg_0.4_Mo_0.7_O_6−δ_ oxides at 600–900 °C in air and argon.

Sr_2_Fe_1.2_Mg_0.2_Mo_0.6_O_6−δ_		600 °C in air	700 °C in air	800 °C in air	900 °C in air
	Oxygen content	**5.86**	**5.85**	**5.83**	**5.82**
		600 °C in argon	700 °C in argon	800 °C in argon	900 °C in argon
	Oxygen content	**5.49**	**5.39**	**5.31**	**5.17**
Sr_2_Fe_0.9_Mg_0.4_Mo_0.7_O_6−δ_		600 °C in air	700 °C in air	800 °C in air	900 °C in air
	Oxygen content	**5.87**	**5.86**	**5.85**	**5.83**
		600 °C in argon	700 °C in argon	800 °C in argon	900 °C in argon
	Oxygen content	**5.57**	**5.48**	**5.39**	**5.28**

**Table 4:** The structural properties, electrical conductivity, TEC values, and redox stability of Sr_2_(Fe,Mo)O_6−δ_-type materials.

Compound	Crystal Structure	Electrical Conductivity [S·cm^−1^]	TEC [×10^−6^ K^−1^]	Redox Stability	References
Sr_2_Fe_1.2_Mg_0.2_Mo_0.6_O_6−δ_	*Fm*-3*m*	56.23–42.66 in air 600–800 °C; 7.88–10.26 in 5% H_2_/Ar 600–800 °C	12.9–14.6 in air; 14.6–16.7 in 5% H_2_/Ar	redox stable	This work
Sr_2_Fe_0.9_Mg_0.4_Mo_0.7_O_6−δ_	*Fm*-3*m*	7.90–7.48 in air 600–800 °C; 0.29–0.34 in 5% H_2_/Ar 600–800 °C	13.5–15.7 in air; 14.2–15.1 in 5% H_2_/Ar	redox stable	This work
Sr_2_Fe_1.5_Mo_0.3_Cu_0.2_O_6−δ_	*Fm*-3*m*	0.06–0.36 in 5% H_2_/Ar at 600–850 °C	-	decomposed in H_2_	[35]
Sr_2_Fe_1.5_Mo_0.5_O_6−δ_	*Fm*-3*m*	2.89–5.55 in 5% H_2_/Ar at 600–850 °C	-	Redox stable	[35]
	*Pm*-3*m*	13 in air at 400–600 °C 50 in 5% H_2_/Ar at 850 °C	13.5–18.3 in air	Redox stable	[28]
SrFe_0.5_Mn_0.25_Mo_0.25_O_3−δ_	*Pm*-3*m*	3 in air at 850 °C 10 in 5% H_2_/Ar at 850 °C	12.9–14.5 in air	Redox stable	[28]
Sr_2_MgMoO_6−δ_	*I*-1	0.8 @800 °C in 5% H_2_/Ar; 0.003 in air @800 °C	-	stable up to 1200 °C in 5% H_2_/Ar	[49]
La_0.5_Sr_0.5_Fe_0.9_Mo_0.1_O_3−__δ_	*Pm*-3*m*	2.7–6.7 in H_2_, 600–800 °C	13.4 in air; 15.1 in 5% H_2_/Ar	stable up to 750 °C in H_2_	[52]
Sr_1.9_Fe_1.5_Mo_0.3_Cu_0.2_O_6−δ_	-	54.8 in air 630 °C	19.39 in air	decomposed in H_2_	[53]
Sr_2_FeMo_2/3_Mg_1/3_O_6−δ_	*Fm*-3*m*	4–5 in air 600–800 °C; 9–13 in H_2_ 600–800 °C	16.9 in air	Redox stable	[38]
Sr_2_Fe_1.3_Ti_0.1_Mo_0.6_O_6−δ_	*Fm*-3*m*	220–160 in 5% H_2_/Ar at 500–800 °C	13.5 in air to 550 °C	Stable in H_2_	[36]
Sr_2_TiFe_0.5_Mo_0.5_O_6−δ_	*Pm*-3*m*	22.3 in H_2_ at 800 °C	11.2 in H_2_	stable in H_2_ and syngas fuels	[54]
Sr_2_Mg_0.95_Al_0.05_MoO_6−δ_	-	5.4 in 5% H_2_/Ar at 800 °C	-	Redox stable	[55]
Sr_2_TiNi_0.5_Mo_0.5_O_6−δ_	-	17.5 in H_2_ at 800 °C	12.8 in air	stable in humidified H2	[37]
Sr_2−x_Ba_x_FeMoO_6−δ_	*I*4/*m* (x = 0); *Fm*-3*m* (others)	100–1000 in 5% H_2_/Ar	13.8 in air (for x = 0)	Stable in 5% H_2_/Ar	[24,25]
Sr_2−x_Ba_x_MnMoO_6−δ_	*P*2_1_/*n* (x = 0); *Fm*-3*m* (others)	0.24–1.41 in 5% H_2_/Ar	11.5–14.8 in air (for x = 0)	Stable in 5% H_2_/Ar	[24,25]
Sr_2−x_Ba_x_MgMoO_6−δ_	*I*4/*m* (x = 0); *Fm*-3*m* (others)	0.14–1.38 in 5% H_2_/Ar	13.8–18.2 in air (for x = 0)	Redox stable	[24,25]
Sr_2_Mg_0.3_Co_0.7_MoO_6−δ_	*I*-1	9–7 in 5% H_2_/Ar at 600–800 °C	13.9 in air	-	[40]
SrFe_0.45_Co_0.45_Mo_0.1_O_3−δ_	*Pm*-3*m*	298 in air at 300 °C	14.8–30.8 in air	Stable in air	[39]

**Table 5:** Structural parameters of Sr_2_Fe_1.2_Mg_0.2_Mo_0.6_O_6−δ_ and Sr_2_Fe_0.9_Mg_0.4_Mo_0.7_O_6−δ_ oxides with Ce_0.8_Gd_0.2_O_1.9_ from 50:50 wt.% mixtures annealed in air for 4 h at 1200 °C.

Composition	Sr_2_Fe_1.2_Mg_0.2_Mo_0.6_O_6−δ_	Ce_0.8_Gd_0.2_O_1.9_	Sr_2_Fe_0.9_Mg_0.4_Mo_0.7_O_6−δ_	Ce_0.8_Gd_0.2_O_1.9_
space group	*Fm*-3*m*	*Fm*-3*m*	*Fm*-3*m*	*Fm*-3*m*
*a* [Å]	7.8567(1)	5.4249(1)	7.8657(1)	5.4256(1)
*V* [Å^3^]	484.98(1)	159.66(1)	486.64(1)	159.71(1)
R_p_ (%)	1.82		2.16	
R_wp_ (%)	2.55		3.29	

**Table 6:** Structural parameters of Sr_2_Fe_1.2_Mg_0.2_Mo_0.6_O_6__−δ_ and Sr_2_Fe_0.9_Mg_0.4_Mo_0.7_O_6__−δ_ oxides with Ce_0.8_Gd_0.2_O_1.9_ from 50:50 wt.% mixtures annealed in 5 vol.% H_2_/Ar at 800 °C for 100 h.

Composition	Sr_2_Fe_1.2_Mg_0.2_Mo_0.6_O_6−δ_	Ce_0.8_Gd_0.2_O_1.9_	Sr_2_Fe_0.9_Mg_0.4_Mo_0.7_O_6−δ_	Ce_0.8_Gd_0.2_O_1.9_
space group	*Fm*-3*m*	*Fm*-3*m*	*Fm*-3*m*	*Fm*-3*m*
*a* [Å]	7.8811(1)	5.4285(1)	7.8801(1)	5.4289(1)
*V* [Å^3^]	489.52(1)	159.97(1)	489.32(1)	160.00(1)
R_p_ (%)	2.05		1.99	
R_wp_ (%)	2.87		2.76	

## Data Availability

The data presented in this study are available on request from the corresponding author.
